# Supplementary material for: Continental drift triggered the Early Permian aridification of North China
Source: Nat Commun. 2025 Jan 4;16:384. doi: 10.1038/s41467-024-55804-8 (PMC11699124; doi:10.1038/s41467-024-55804-8)
Supplement: Supplementary file 1 — Supplementary Information [file 41467_2024_55804_MOESM1_ESM.pdf]

Supplementary Materials for

Continental drift triggered the Early Permian aridification of North China

Qiang Ren[1,2,3*], Shihong Zhang[2*], Mingcai Hou[1,3*], Dongyu Zheng[1,3], Huaichun Wu[2], Tianshui Yang[2], Haiyan Li[2], Anqing Chen[1,3], and James G. Ogg[1,3,4]

[1] *State Key Laboratory of Oil and Gas Reservoir Geology and Exploitation, Chengdu University of Technology, Chengdu 610059, China.*
[2] *State Key Laboratory of Biogeology and Environmental Geology, China University of Geosciences, Beijing 100083, China.*
[3] *Key Laboratory of Deep-time Geography and Environment Reconstruction and Applications, Chengdu University of Technology, Chengdu 610059, China.*
[4] *Department of Earth, Atmospheric, and Planetary Sciences, Purdue University, West Lafayette, IN 47907-2051, USA.*

*Corresponding author. Qiang Ren (Email: renqiang@cdut.edu.cn)
Shihong Zhang (Email: shzhang@cugb.edu.cn)
Mingcai Hou (Email: houmc@cdut.edu.cn)

This file includes:

Supplementary Text
Supplementary figures (Figs. 1 to 12)
Supplementary tables (Tables 1 to 5)
Supplementary references

SUPPLEMENTARY TEXT

Analysis of the Permian-Early Triassic paleomagnetic poles in North China

The previously reported Early Permian paleomagnetic data from the Ordos Basin in western North China have been excluded due to the lack of reliability tests[1]. For the Middle to Late Permian paleomagnetic data, we followed Ren et al.[2], utilizing reliable paleomagnetic poles from various formations: the volcanic rocks of the Elitu and Sanmianjin formations (283-266 Ma; ref. 3), the andesites of the Yujiabeigou Formation (~270-260 Ma; ref. 4), middle Permian clastic rocks[5], and member 3 of the Qingfengshan Formation volcanic rocks (~255 Ma). Based on the analysis of Zhou et al.[6], we account for significant inclination shallowing in the Early Triassic paleomagnetic data[5,7-11] obtained from the clastic rocks in the Ordos Basin. Accordingly, we utilized the Early Triassic paleomagnetic mean pole from these seven studies, applying an inclination flattening correction factor (f = 0.6).

We compared our new 290 Ma and 280 Ma poles with these Early Permian to Early Triassic paleomagnetic poles. The Middle Permian to Early Triassic poles differ significantly from our new poles. However, our 280 Ma pole closely aligns with the pole from the volcanic rocks of the Elitu and Sanmianjin formations (283-266 Ma; ref. 3). The ~6 My age difference between these two poles indicates that there was no significant plate movement in North China during this interval.

SUPPLEMENTARY FIGURES

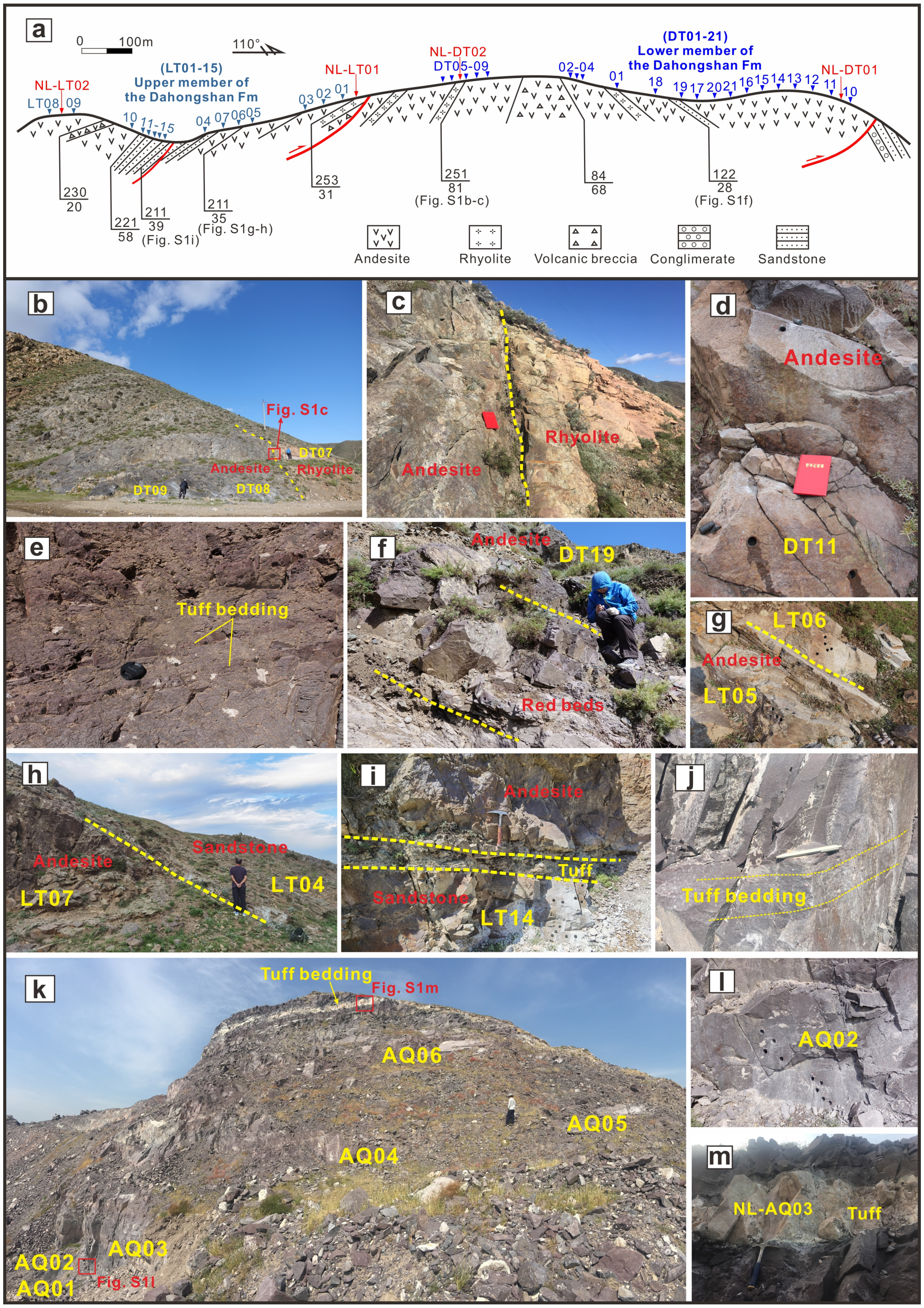

Supplementary Fig. 1. Sketch section and photographs of sampling outcrops. (a) The sketch section of the Dahongshan Formation near Datan village of south Siziwangqi. The volcanic rocks interbedded with volcano-clastic rocks from the Lower member (b–f) and Upper member (g-i) of the Dahongshan Formation. (j–m) The red beds with tuff interlayer from member 1 of the Qingfengshan Formation in Anqing of the northeast Chifeng. Fm = Formation.

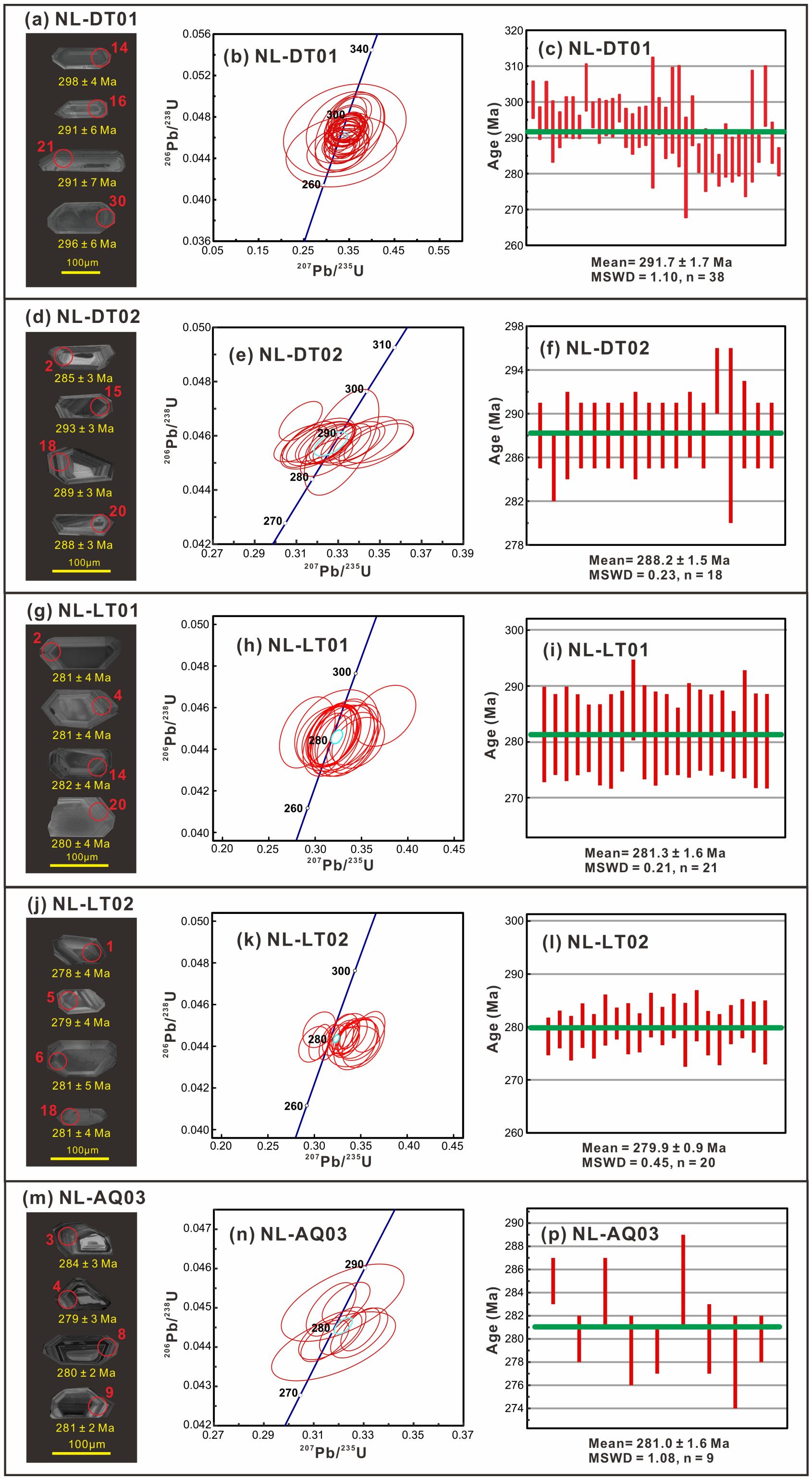

Supplementary Fig. 2. The U-Pb dating results. (a, d, g, j and m) Cathodoluminescence images of representative zircon grains and corresponding $^{206}Pb/^{238}U$ ages of individual analyzed spots. (b, e, h, k and n) U–Pb concordia diagrams of zircon grains. (c, f, i, l and p) Bar plots showing weighted mean $^{206}Pb/^{238}U$ ages.

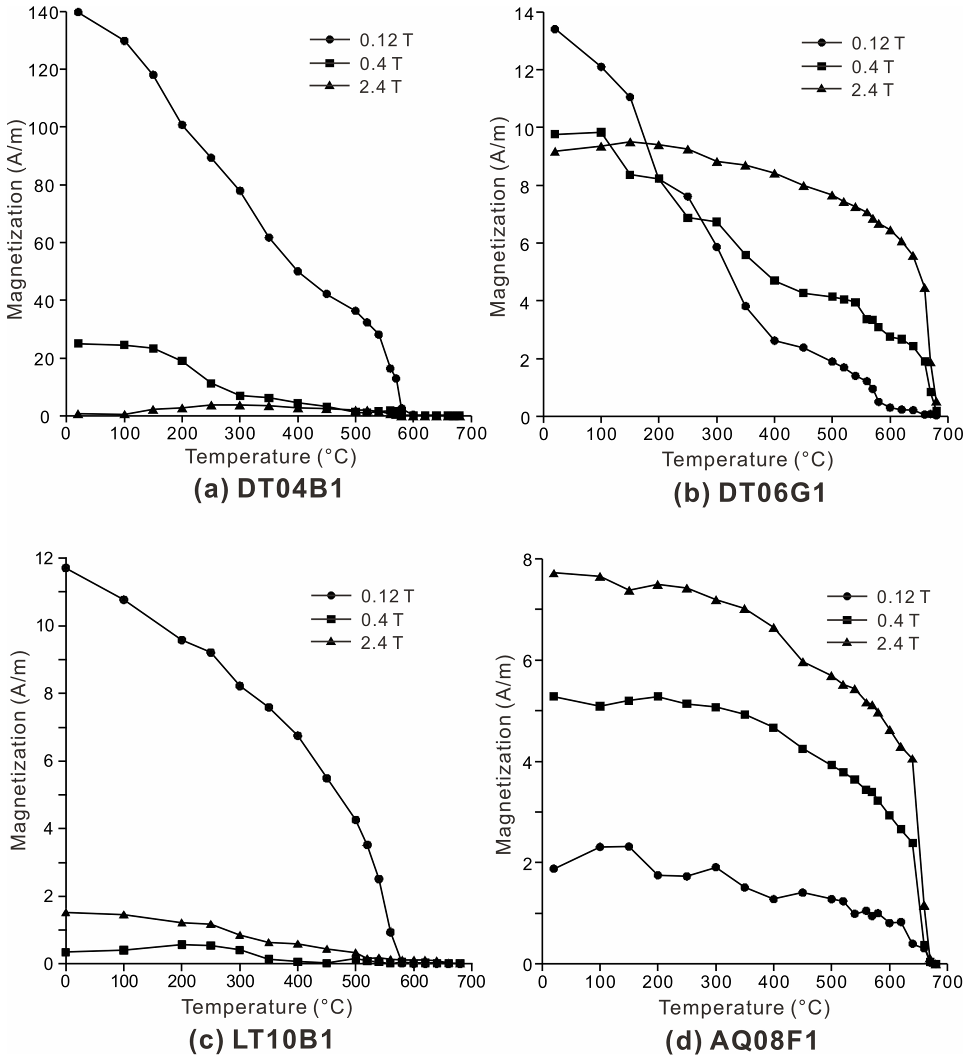

Supplementary Fig. 3. Rock magnetic results. Stepwise thermal demagnetization of the three orthogonal IRM components for representative samples. (a and b) Lower member of the Dahongshan Formation volcanic rocks. (c) Upper member of the Dahongshan Formation volcanic rocks. (d) Member 1 of the Qingfengshan Formation red siltstones.

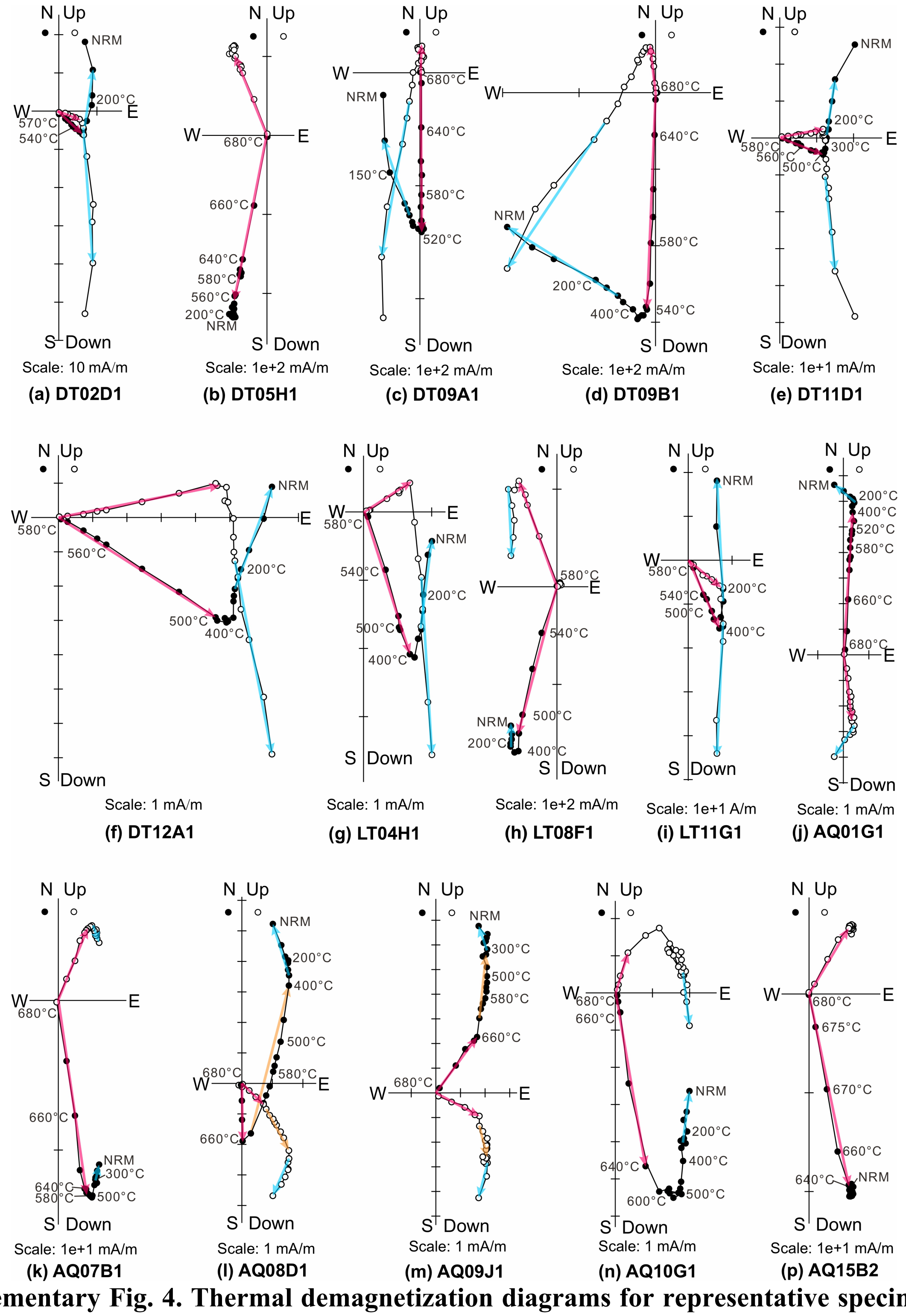

Supplementary Fig. 4. Thermal demagnetization diagrams for representative specimens in geographic coordinates. Solid/open symbols of the orthogonal plots represent projection onto the horizontal/vertical plane. Red arrow, high-temperature component; Orange arrow, mid-temperature component; Blue arrow, low-temperature component (a–f) Lower member of the Dahongshan Formation volcanic rocks. (g–i) Upper member of the Dahongshan Formation volcanic rocks and sandstones. (j-p) Member 1 of the Qingfengshan Formation red siltstones.

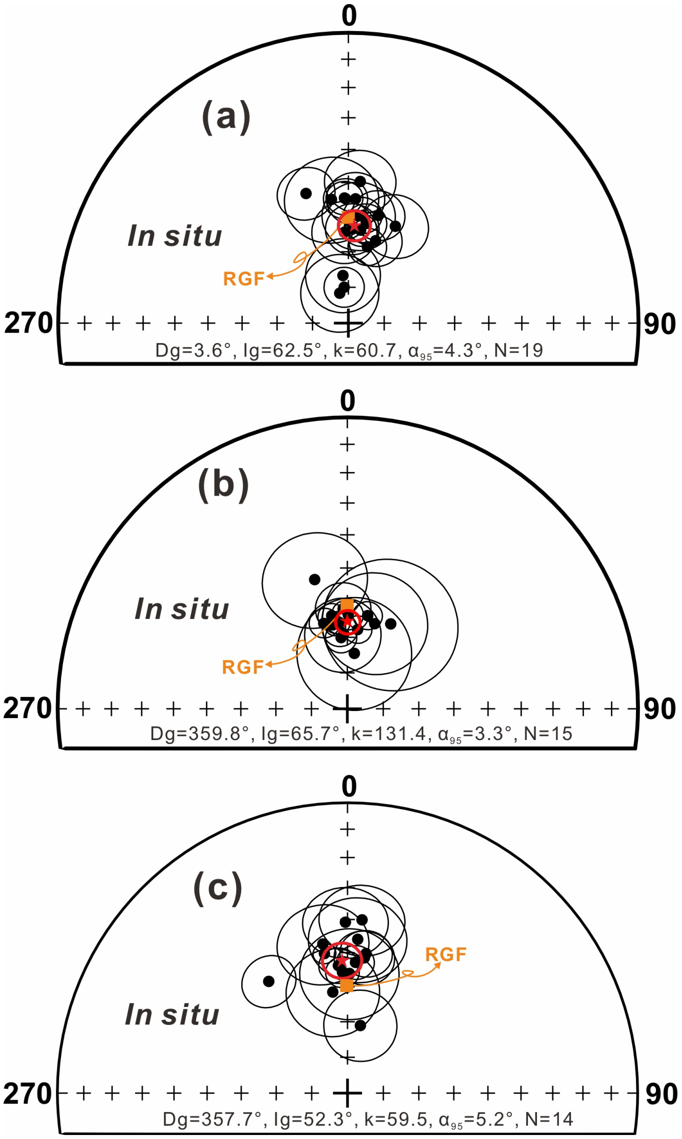

Supplementary Fig. 5. Equal-area stereographic projections of the site-mean directions of the low temperature components. Lower (upper) hemisphere directions are represented by solid (open) symbols. (a) Lower member of the Dahongshan Formation, (b) Upper member of the Dahongshan Formation, and (c) Member 1 of the Qingfengshan Formation. RGF = recent geomagnetic field.

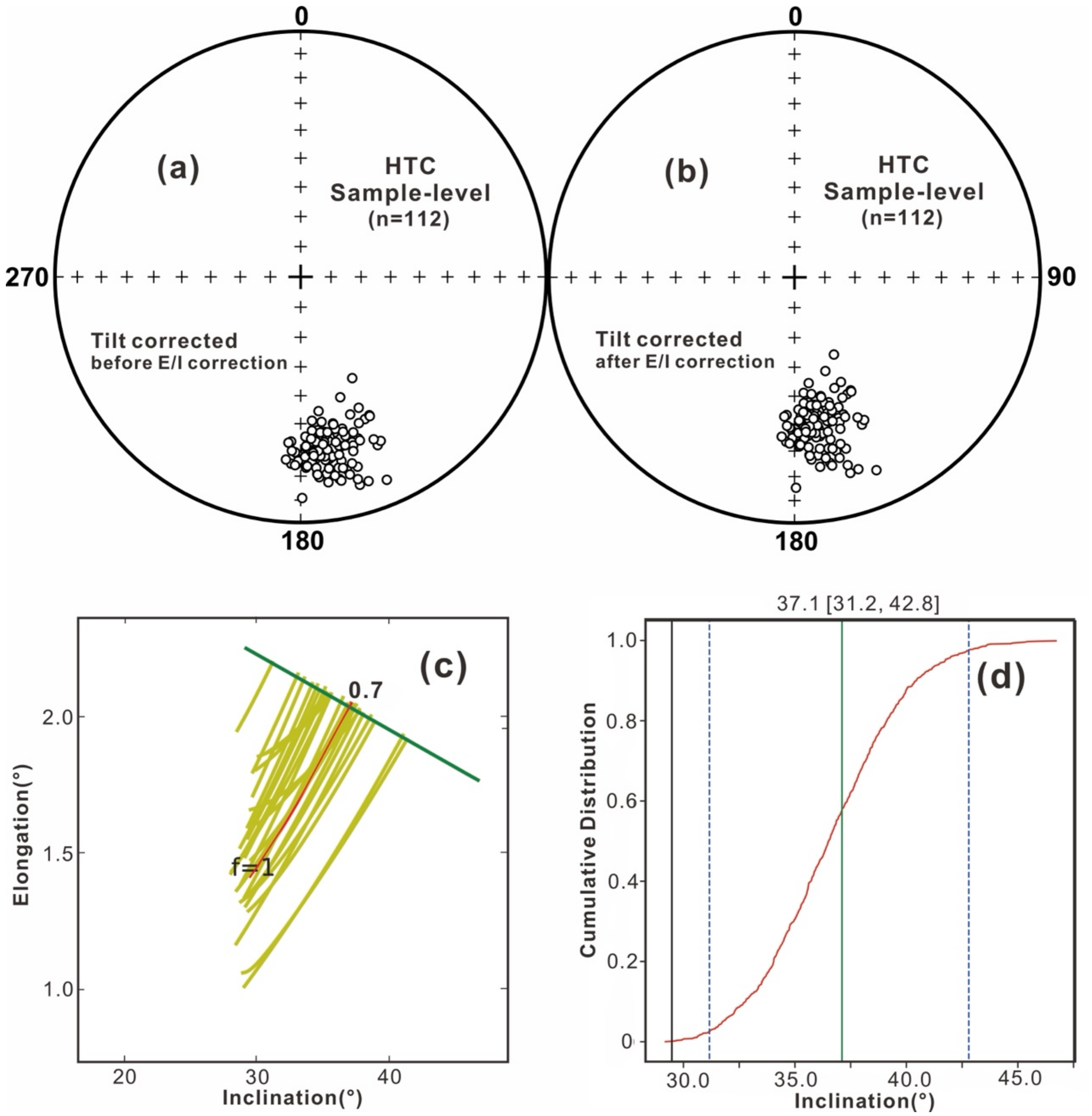

Supplementary Fig. 6. The Elongation/inclination (E/I) correction for member 1 of the Qingfengshan Formation siltstones. (a) Equal area projections of the HTC directions in stratigraphic coordinates. (b) Results after the E/I correction[12], plotted at sample level, N=112. (c) Plot of elongation vs. inclination as a function of flattening factor ("*f*" value). (d) Cumulative distribution of the corrected inclinations.

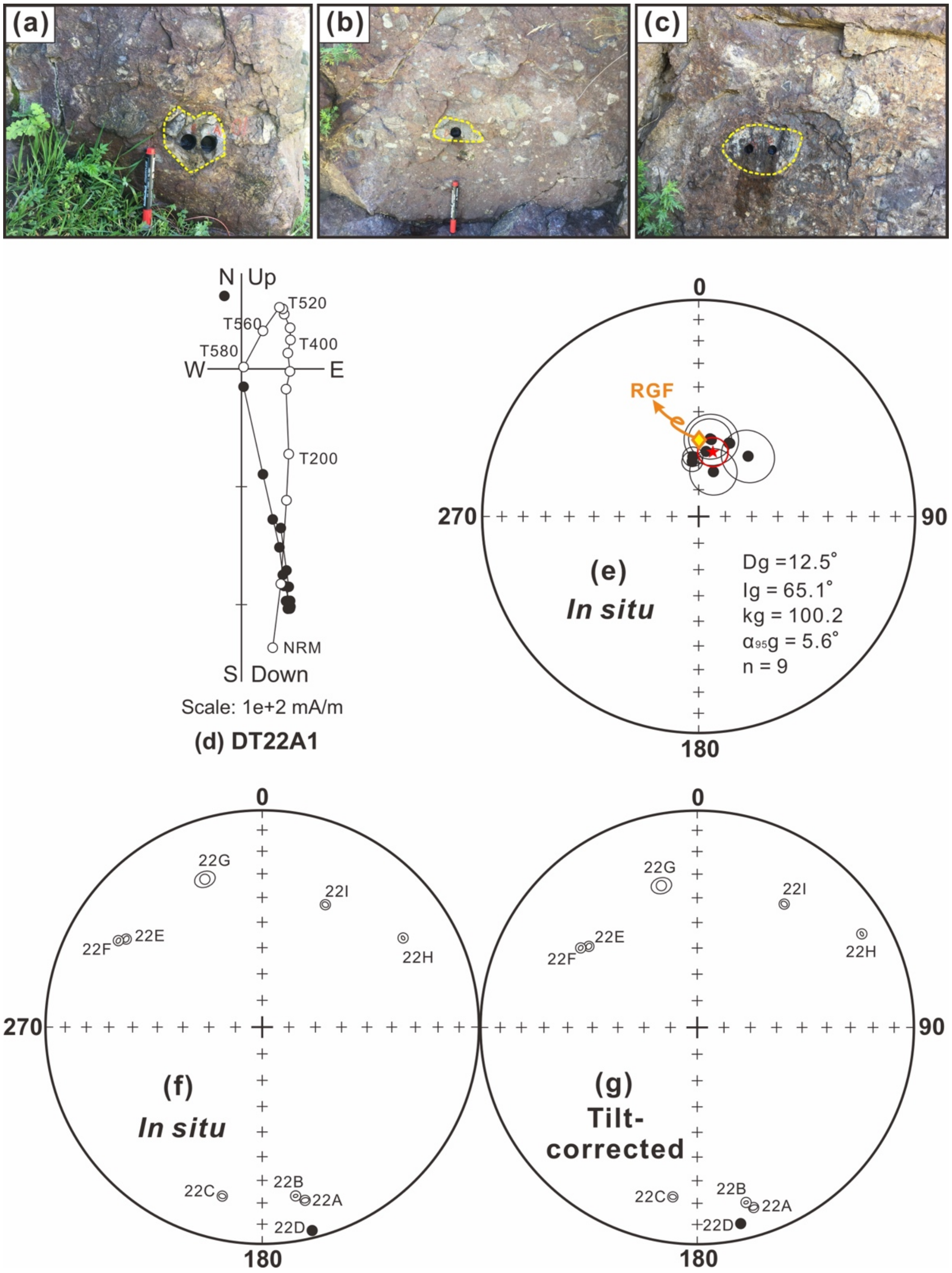

Supplementary Fig. 7. Conglomerate test results and corresponding photographs of sampling outcrops from the Lower member of the Dahongshan Formation. (a-c) Photographs of the volcanic breccia of sampling outcrops. (d) Orthogonal projection diagrams for representative specimens in geographic coordinates. (e) Equal-area projection of the LTC directions. (f-g) Equal-area projections of the HTC directions.

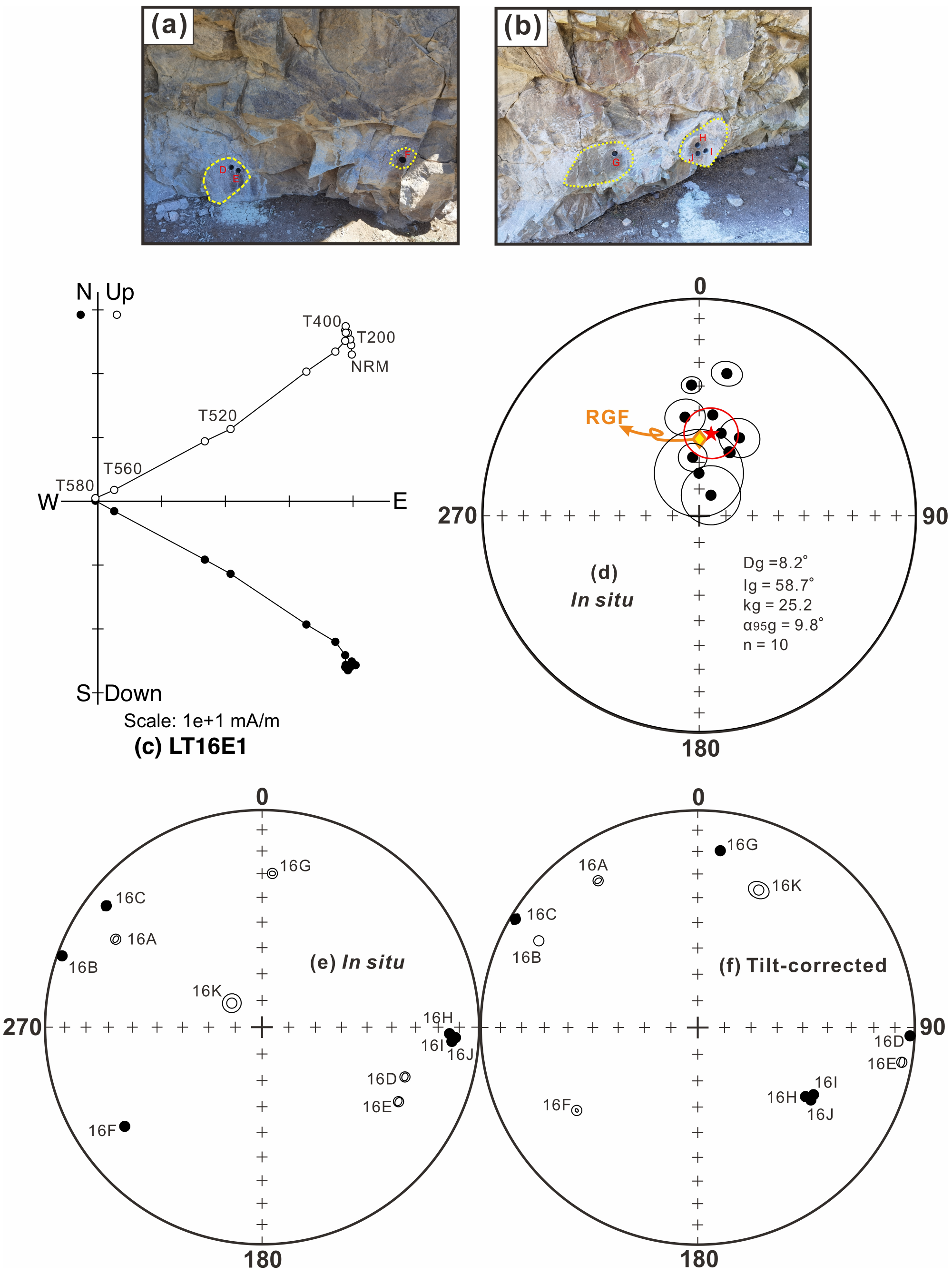

Supplementary Fig. 8. Conglomerate test results and corresponding photographs of sampling outcrops from the Upper member of the Dahongshan Formation. (a-b) Photographs of the volcanic breccia of sampling outcrops. (c) Orthogonal projection diagrams for representative specimens in geographic coordinates. (d) Equal-area projection of the LTC directions. (e-f) Equal-area projections of the HTC directions.

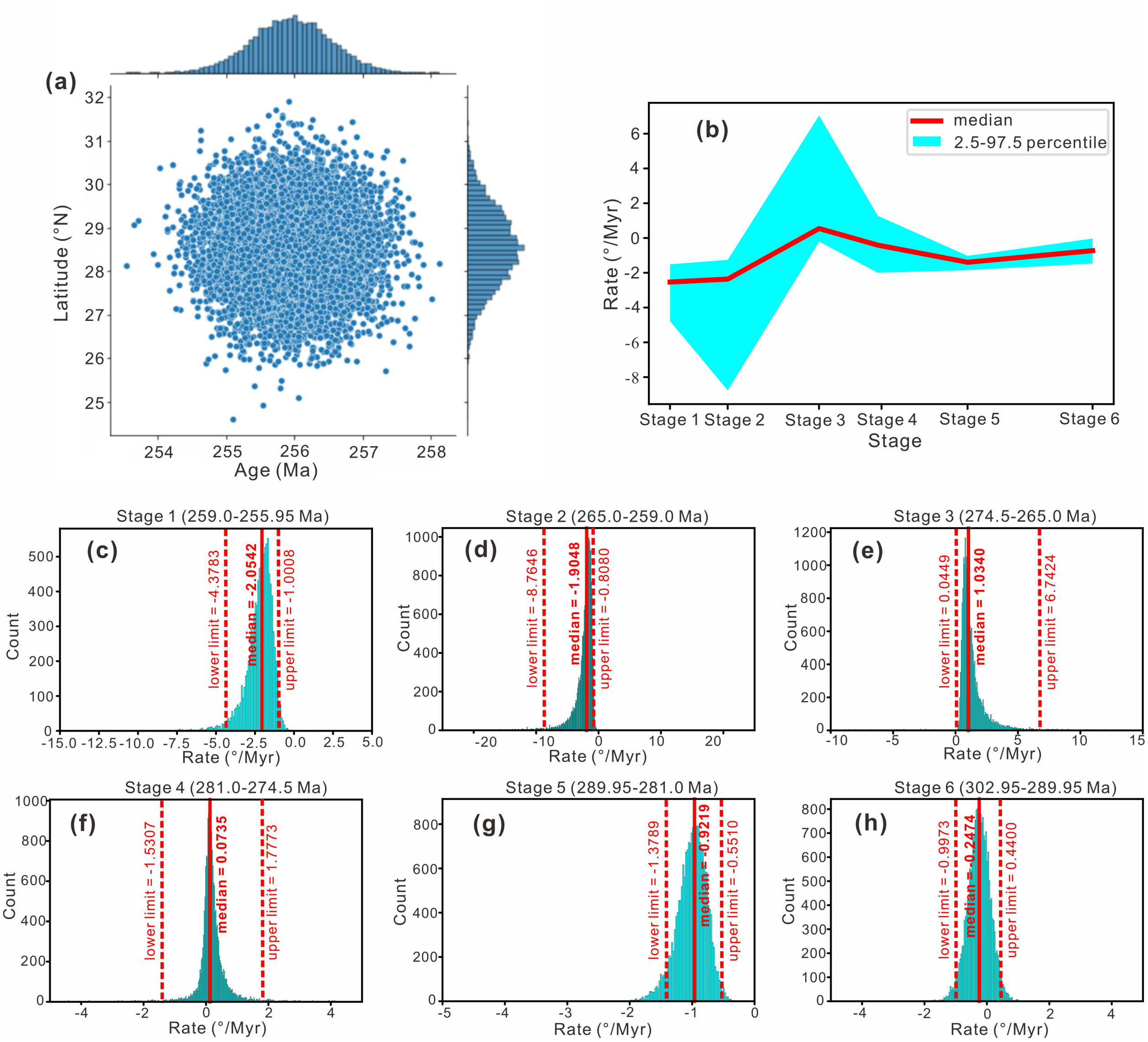

Supplementary Fig. 9. The simulation of latitudinal motion rate of the North China Block (NCB). (a) Resampled data points after 10000 simulations (an example from 255.95 Ma). (b) The motion rate of the NCB at 6 stages. Regarding each time period, a total of 10000 motion rates were calculated using the Monte Carlo simulation. (c-h) The count estimation of the motion rate distribution of the NCB from 10000 times of the Monte Carlo simulation. Negative values represent northward movements; positive values represent southward movements.

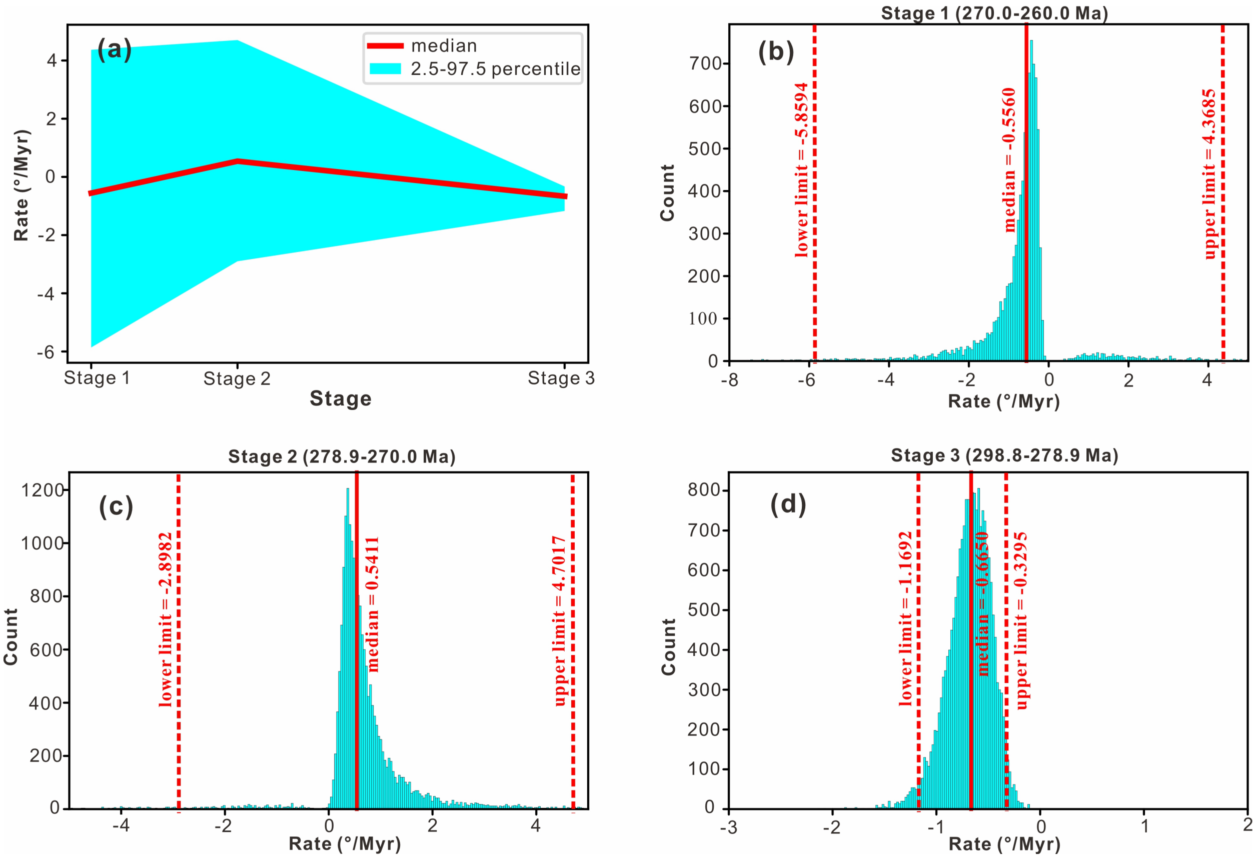

Supplementary Fig. 10. The simulation of latitudinal motion rate of the South China Block (SCB). (a) The motion rate of the SCB at 3 stages. Regarding each time period, a total of 10000 motion rates were calculated using the Monte Carlo simulation. (b-d) The count estimation of the motion rate distribution of the SCB from 10000 times of the Monte Carlo simulation. Negative values represent northward movements; positive values represent southward movements.

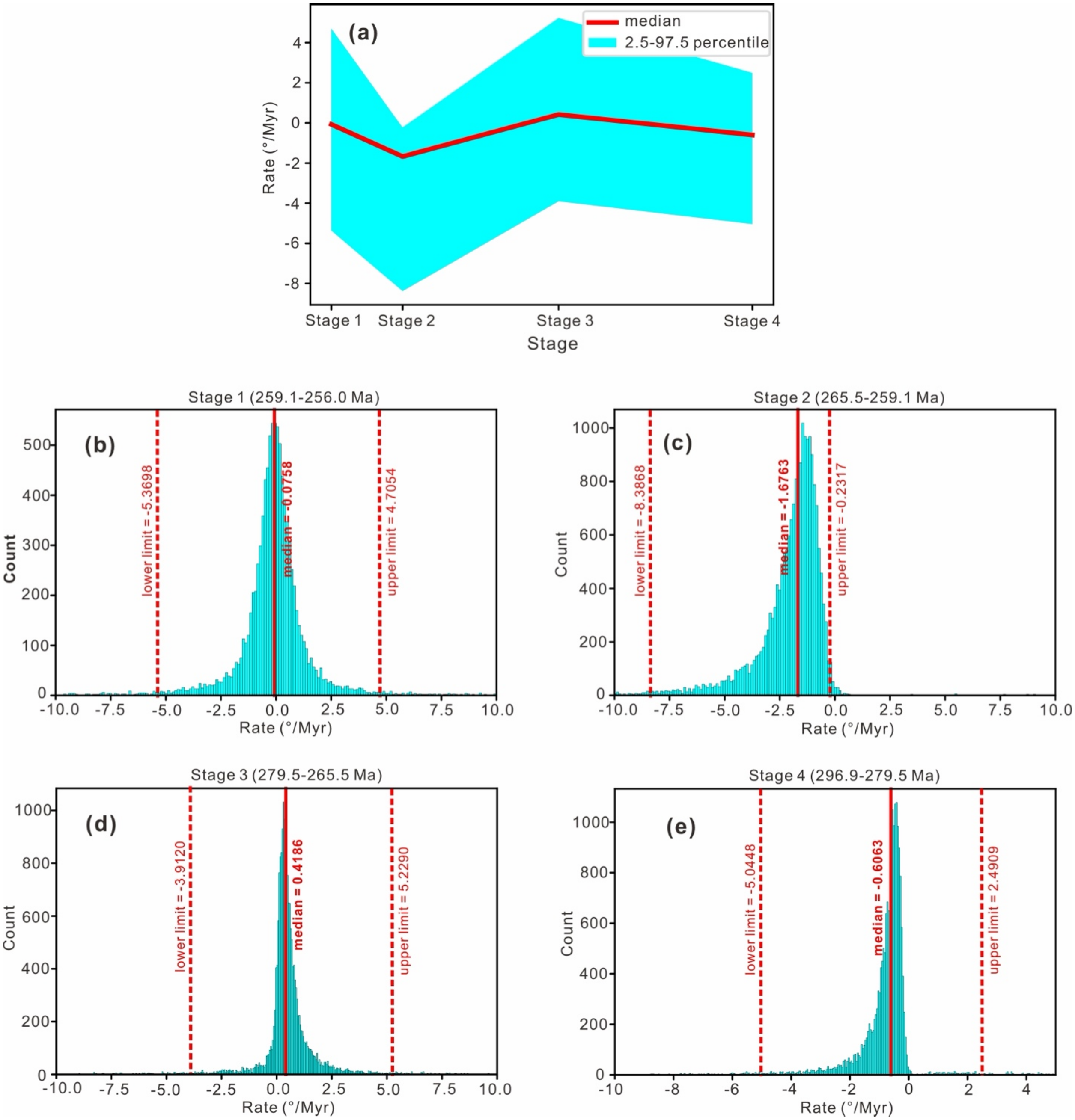

Supplementary Fig. 11. The simulation of latitudinal motion rate of the North Qiangtang Block (NQT). (a) The motion rate of the NQT at 4 stages. Regarding each time period, a total of 10000 motion rates were calculated using Monte Carlo simulation. (b-e) The count estimation of the motion rate distribution of the NQT from 10000 times of Monte Carlo simulation. Negative values represent northward movements; positive values represent southward movements.

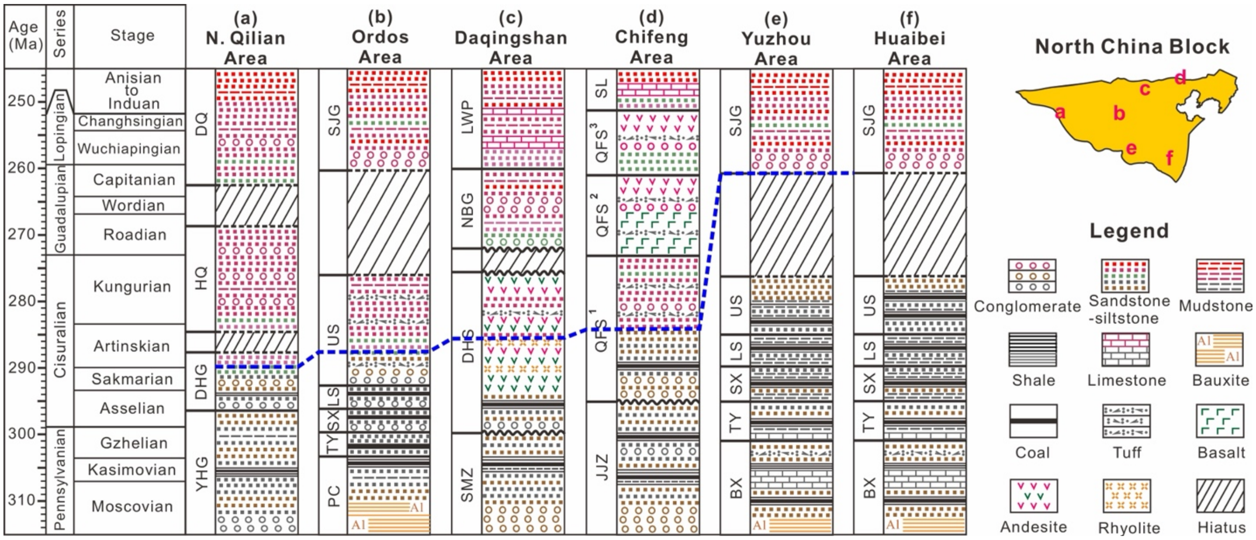

Supplementary Fig. 12. Late Carboniferous-Middle Triassic stratigraphic columns of the typical section in the NCB. (a-f) different stratigraphic columns, corresponding to the alphabetic numbers of different locations in the NCB. The dashed blue line represents the beginning of the red beds. The stratigraphic information is cited from Wu et al.[13] and Shen et al.[14].

SUPPLEMENTARY TABLES

Supplementary Table 1.

Summary of the zircon U-Pb data for the Dahongshan Formation and member 1 of the Qingfengshan Formation.

Spot	Total Pb	^{232}Th	^{238}U	Th/U	Isotope ratio						Isotopic age (Ma)					
	10^{-6}	10^{-6}	10^{-6}		$^{207}Pb/^{206}Pb$	1σ	$^{207}Pb/^{235}U$	1σ	$^{206}Pb/^{238}U$	1σ	$^{207}Pb/^{206}Pb$	1σ	$^{207}Pb/^{235}U$	1σ	$^{206}Pb/^{238}U$	1σ
**Lower member of the Dahongshan Formation andesite (NL-DT01)**																
1	8.24	124.13	165.65	0.75	0.054114	0.004322	0.348009	0.024897	0.047727	0.000838	376	181	303	19	301	5
2	11.48	133.26	246.73	0.54	0.054712	0.003056	0.351385	0.019673	0.046667	0.00073	467	94	306	15	294	4
3	10.58	149.58	215.00	0.70	0.053499	0.004994	0.344829	0.029602	0.047384	0.00117	350	213	301	22	298	7
4	3.76	66.89	74.64	0.90	0.057806	0.00693	0.348636	0.035138	0.04629	0.001384	524	265	304	26	292	9
5	9.04	124.19	188.86	0.66	0.052967	0.003604	0.340315	0.023459	0.046368	0.000806	328	156	297	18	292	5
6	6.56	87.41	135.97	0.64	0.055606	0.004048	0.344905	0.023152	0.046935	0.000917	435	163	301	17	296	6
7	8.73	99.30	186.80	0.53	0.054469	0.003766	0.350022	0.023641	0.046912	0.000939	391	156	305	18	296	6
8	16.61	347.28	307.90	1.13	0.054083	0.002197	0.343433	0.01306	0.046497	0.000517	376	93	300	10	293	3
9	10.17	129.78	202.33	0.64	0.055498	0.004712	0.358345	0.027195	0.048288	0.001068	432	190	311	20	304	7
10	15.10	245.99	289.22	0.85	0.054647	0.002756	0.352929	0.017618	0.04704	0.000581	398	113	307	13	296	4
11	10.64	133.66	221.57	0.60	0.053836	0.005135	0.342654	0.031591	0.046767	0.001007	365	182	299	24	295	6
12	11.06	123.46	230.76	0.54	0.053313	0.004157	0.349172	0.027844	0.046898	0.000794	343	178	304	21	295	5
13	6.57	96.70	134.56	0.72	0.053728	0.003693	0.343635	0.023583	0.046915	0.000868	367	156	300	18	296	5
14	11.61	159.45	241.11	0.66	0.05338	0.002678	0.347141	0.017641	0.047339	0.000619	346	113	303	13	298	4
15	12.25	289.48	222.17	1.30	0.054092	0.003979	0.33847	0.023487	0.046451	0.000881	376	160	296	18	293	5
16	10.11	208.39	194.00	1.07	0.053468	0.004521	0.334963	0.026583	0.046172	0.000913	350	191	293	20	291	6
17	8.50	113.38	178.35	0.64	0.052759	0.003914	0.337519	0.02529	0.046482	0.000906	317	170	295	19	293	6
18	6.61	91.29	139.75	0.65	0.056209	0.00438	0.34973	0.024831	0.046548	0.000883	461	177	305	19	293	5
19	3.98	52.55	86.77	0.61	0.054469	0.014617	0.340374	0.089465	0.0467	0.002954	391	511	297	68	294	18
20	12.21	173.53	255.76	0.68	0.053301	0.004078	0.344332	0.026666	0.046983	0.000834	343	169	300	20	296	5
21	5.93	78.02	128.75	0.61	0.057328	0.005228	0.351537	0.027753	0.046221	0.001153	506	197	306	21	291	7
22	5.82	90.80	118.69	0.76	0.057865	0.007221	0.357798	0.040995	0.047255	0.001941	524	271	311	31	298	12
23	5.17	72.68	108.85	0.67	0.056209	0.010385	0.342017	0.057356	0.046983	0.002288	461	372	299	43	296	14
24	2.80	44.61	63.15	0.71	0.052992	0.013584	0.332914	0.080859	0.044668	0.002263	328	505	292	62	282	14
25	5.54	72.24	120.49	0.60	0.054995	0.003998	0.348244	0.023041	0.046777	0.001121	413	165	303	17	295	7
26	11.34	158.77	246.67	0.64	0.054779	0.002759	0.336411	0.016111	0.045078	0.000638	467	118	294	12	284	4
27	10.19	183.51	205.28	0.89	0.05467	0.006022	0.333645	0.038874	0.044997	0.001411	398	248	292	30	284	9
28	7.87	118.19	166.53	0.71	0.053895	0.004172	0.331132	0.024707	0.045349	0.000917	365	171	290	19	286	6
29	9.77	145.51	212.98	0.68	0.054224	0.003613	0.329685	0.021911	0.044539	0.000703	389	145	289	17	281	4
30	8.90	117.52	189.40	0.62	0.054651	0.004995	0.342705	0.030909	0.045433	0.001189	398	201	299	23	286	7
31	9.97	138.86	212.23	0.65	0.056591	0.004529	0.339456	0.022302	0.045018	0.001006	476	178	297	17	284	6
32	8.91	132.19	186.42	0.71	0.055016	0.005578	0.34225	0.034561	0.045319	0.001039	413	232	299	26	286	6
33	5.04	61.73	113.09	0.55	0.051678	0.007091	0.321071	0.042567	0.044751	0.0014	272	294	283	33	282	9
34	10.76	159.32	218.33	0.73	0.05429	0.007209	0.350753	0.050136	0.046529	0.002522	383	302	305	38	293	16
35	11.47	150.92	242.76	0.62	0.052753	0.002253	0.330174	0.014067	0.045513	0.000607	317	98	290	11	287	4

36	7.94	100.49	161.32	0.62	0.053379	0.004751	0.346114	0.02789	0.047897	0.001369	346	234	302	21	302	8
37	10.02	117.41	213.71	0.55	0.055389	0.004479	0.342102	0.027169	0.045778	0.000928	428	181	299	21	289	6
38	7.34	76.43	164.54	0.46	0.055965	0.002663	0.340465	0.01534	0.044922	0.000622	450	106	298	12	283	4
**Lower member of the Dahongshan Formation rhyolite (NL-DT02)**																
1	15.73	147.66	170.91	0.86	0.05176	0.00127	0.32616	0.00778	0.04569	0.00048	275	35	287	6	288	3
2	12.36	112.47	148.42	0.76	0.0521	0.00139	0.32451	0.0083	0.0452	0.00046	290	40	285	6	285	3
3	6.89	55.59	87.89	0.63	0.05562	0.002	0.34825	0.01178	0.04569	0.00059	437	52	303	9	288	4
4	13.65	122.45	157.89	0.78	0.05196	0.00127	0.32858	0.00823	0.04571	0.00046	284	39	288	6	288	3
5	7.81	74.47	87.36	0.85	0.05329	0.00179	0.33522	0.01138	0.04572	0.00053	341	56	294	9	288	3
6	12.18	106.07	136.00	0.78	0.05164	0.00198	0.32443	0.00884	0.0457	0.00049	270	43	285	7	288	3
7	6.76	51.13	82.60	0.62	0.05559	0.00185	0.34948	0.01102	0.04572	0.00048	436	52	304	8	288	3
8	43.39	164.01	160.43	1.02	0.04768	0.00234	0.32018	0.01181	0.04571	0.00062	83	59	282	9	288	4
9	8.61	71.02	108.98	0.65	0.05044	0.00146	0.31634	0.00865	0.04568	0.00056	215	41	279	7	288	3
10	16.60	149.44	180.66	0.83	0.05301	0.0023	0.3337	0.0079	0.04572	0.00048	329	34	292	6	288	3
11	8.02	68.67	97.53	0.70	0.0511	0.00174	0.32147	0.01072	0.04571	0.00054	245	55	283	8	288	3
12	18.88	153.46	201.45	0.76	0.051	0.00252	0.32077	0.0118	0.04577	0.00049	241	65	282	9	289	3
13	7.52	64.39	91.96	0.70	0.05155	0.00147	0.32325	0.00907	0.04574	0.00051	266	44	284	7	288	3
14	24.98	265.32	227.51	1.17	0.04815	0.00181	0.31205	0.00686	0.04654	0.00048	107	32	276	5	293	3
15	30.78	204.80	229.81	0.89	0.05185	0.00288	0.33164	0.0118	0.04576	0.00129	279	37	291	9	288	8
16	10.97	78.98	133.33	0.59	0.0544	0.00206	0.34204	0.01094	0.0458	0.0006	388	48	299	8	289	4
17	16.05	146.19	182.42	0.80	0.05376	0.00119	0.33873	0.00741	0.04572	0.00042	361	33	296	6	288	3
18	14.53	139.97	159.64	0.88	0.04862	0.00135	0.31209	0.0085	0.04572	0.00045	130	45	276	7	288	3
**Upper member of the Dahongshan Formation rhyolite (NL-LT01)**																
1	7.63	72.90	124.53	0.59	0.05236	0.00218	0.31960	0.01287	0.04465	0.00069	302	92	282	10	282	4
2	13.99	149.37	226.61	0.66	0.05182	0.00158	0.31975	0.01023	0.04460	0.00060	276	70	282	8	281	4
3	9.68	98.06	155.88	0.63	0.05384	0.00252	0.32765	0.01482	0.04456	0.00067	365	106	288	11	281	4
4	9.50	105.37	147.83	0.71	0.05161	0.00205	0.31565	0.01217	0.04458	0.00063	333	95	279	9	281	4
5	22.68	311.19	308.36	1.01	0.05127	0.00125	0.31473	0.00784	0.04453	0.00053	254	57	278	6	281	3
6	11.34	124.31	176.92	0.70	0.05561	0.00176	0.34034	0.01137	0.04427	0.00062	435	70	297	9	279	4
7	6.59	60.01	112.56	0.53	0.05227	0.00215	0.31806	0.01284	0.04444	0.00074	298	88	280	10	280	5
8	9.21	85.56	160.18	0.53	0.05228	0.00186	0.31978	0.01119	0.04465	0.00056	298	81	282	9	282	3
9	11.85	103.45	191.64	0.54	0.05975	0.00219	0.37495	0.01383	0.04559	0.00059	594	75	323	10	287	4
10	10.38	97.97	182.85	0.54	0.04741	0.00199	0.29161	0.01226	0.04476	0.00073	78	91	260	10	282	5
11	12.53	117.77	218.91	0.54	0.05182	0.00143	0.31712	0.00921	0.04445	0.00066	276	63	280	7	280	4
12	6.03	63.55	100.05	0.64	0.05271	0.00215	0.31983	0.01216	0.04462	0.00063	317	93	282	9	281	4
13	13.23	125.60	228.24	0.55	0.05226	0.00143	0.31920	0.00886	0.04436	0.00051	298	68	281	7	280	3
14	8.04	83.12	131.95	0.63	0.05371	0.00189	0.32990	0.01227	0.04474	0.00071	367	80	289	9	282	4
15	11.97	132.70	188.11	0.71	0.05596	0.00181	0.34297	0.01132	0.04472	0.00060	450	72	299	9	282	4
16	8.79	105.10	133.74	0.79	0.05121	0.00226	0.31313	0.01353	0.04458	0.00061	250	102	277	10	281	4
17	7.74	78.81	129.49	0.61	0.05217	0.00197	0.32001	0.01158	0.04472	0.00062	300	82	282	9	282	4
18	11.90	133.34	185.52	0.72	0.05683	0.00195	0.34650	0.01160	0.04429	0.00051	483	81	302	9	279	3
19	5.59	57.11	88.17	0.65	0.05224	0.00287	0.32289	0.01841	0.04491	0.00079	295	126	284	14	283	5
20	5.02	44.56	89.00	0.50	0.05418	0.00246	0.32858	0.01459	0.04439	0.00069	389	102	288	11	280	4

21	5.93	61.17	98.15	0.62	0.05534	0.00230	0.33497	0.01339	0.04442	0.00073	433	93	293	10	280	5
**Upper member of the Dahongshan Formation andesite (NL-LT02)**																
1	9.07	76.36	168.63	0.45	0.05218	0.00162	0.31643	0.00981	0.04407	0.00057	300	70	279	8	278	3
2	13.53	145.57	220.65	0.66	0.05265	0.00166	0.32151	0.01040	0.04432	0.00060	322	72	283	8	280	4
3	7.22	64.77	126.69	0.51	0.04928	0.00214	0.29973	0.01349	0.04415	0.00071	161	102	266	11	278	4
4	8.59	80.21	148.92	0.54	0.05438	0.00210	0.33246	0.01298	0.04443	0.00069	387	87	291	10	280	4
5	12.56	169.90	181.91	0.93	0.05012	0.00159	0.30608	0.00975	0.04424	0.00065	211	74	271	8	279	4
6	5.60	61.64	86.18	0.72	0.05576	0.00316	0.33574	0.01742	0.04460	0.00084	443	121	294	13	281	5
7	15.56	166.45	253.34	0.66	0.05515	0.00157	0.33718	0.00913	0.04446	0.00053	417	63	295	7	280	3
8	5.07	54.79	81.15	0.68	0.05811	0.00340	0.34709	0.01805	0.04431	0.00076	600	95	303	14	280	5
9	9.97	118.21	154.83	0.76	0.05488	0.00185	0.33427	0.01168	0.04415	0.00063	406	74	293	9	279	4
10	7.59	80.54	116.78	0.69	0.04955	0.00236	0.30515	0.01429	0.04485	0.00072	172	111	270	11	283	4
11	13.90	144.28	230.34	0.63	0.05405	0.00161	0.33054	0.00957	0.04445	0.00062	372	67	290	7	280	4
12	6.50	63.20	107.60	0.59	0.05472	0.00235	0.33155	0.01262	0.04466	0.00069	467	96	291	10	282	4
13	3.46	36.26	52.79	0.69	0.05222	0.00350	0.34439	0.01906	0.04408	0.00096	295	121	300	14	278	6
14	9.91	117.92	136.72	0.86	0.05286	0.00255	0.35858	0.01434	0.04471	0.00078	324	109	311	11	282	5
15	25.62	440.80	311.04	1.42	0.05212	0.00148	0.31611	0.00905	0.04420	0.00068	300	60	279	7	279	4
16	5.40	62.10	79.06	0.79	0.05628	0.00377	0.34232	0.02315	0.04403	0.00082	465	148	299	18	278	5
17	15.35	192.63	227.29	0.85	0.05516	0.00184	0.33578	0.01007	0.04437	0.00058	420	79	294	8	280	4
18	39.75	636.06	473.78	1.34	0.05493	0.00138	0.33818	0.00884	0.04457	0.00061	409	56	296	7	281	4
19	3.02	39.56	41.16	0.96	0.05689	0.00280	0.34560	0.01636	0.04438	0.00084	487	107	301	12	280	5
20	0.89	11.88	9.87	1.20	0.05386	0.00344	0.35279	0.01862	0.04415	0.00098	365	144	307	14	279	6
**Member 1 of the Qingfengshan Formation tuff (NL-AQ03)**																
1	22.49	233.65	227.21	1.03	0.05177	0.0021	0.32298	0.00634	0.04512	0.00035	275	31	284	5	285	2
2	24.04	217.35	288.78	0.75	0.05348	0.00168	0.3287	0.00519	0.04445	0.00036	349	21	289	4	280	2
3	56.78	591.67	572.34	1.03	0.05099	0.00101	0.31699	0.0066	0.04496	0.00045	240	30	280	5	284	3
4	16.68	162.50	189.33	0.86	0.05172	0.00171	0.31664	0.00838	0.0443	0.00041	273	44	279	6	279	3
5	34.21	437.41	269.01	1.63	0.05162	0.00107	0.3145	0.00636	0.04416	0.00035	269	32	278	5	279	2
6	6.32	69.24	54.68	1.27	0.05217	0.003	0.32159	0.01624	0.04513	0.00065	293	89	283	12	285	4
7	55.87	795.27	348.39	2.28	0.05363	0.00191	0.32822	0.00757	0.04436	0.00042	356	35	288	6	280	3
8	9.54	79.71	120.96	0.66	0.05239	0.00279	0.31796	0.0164	0.04402	0.00058	302	124	280	13	278	4
9	40.64	416.23	422.97	0.98	0.05233	0.0015	0.32161	0.00614	0.04441	0.0004	300	27	283	5	280	2

Supplementary Table 2.

Site-mean values and statistical parameters for the high temperature components from lower Permian volcanic rocks from the Dahongshan Fm and red siltstones from member 1 of the Qingfengshan Fm in the northern North China Block.

Site	n/N	Strike/Dip (°)	Dg (°)	Ig (°)	Ds (°)	Is (°)	k (°)	α_{95} (°)	Plat (°N)	Plon (°E)	A_{95} (°)
**Lower member of the Dahongshan Formation** (~290 Ma) volcanic rocks											
GPS: 41.21°N, 112.22°E (DT01-04, DT10-21); GPS: 41.22°N, 112.20°E (DT05-09)											
DT01	7/7	32/28	127.8	-17.4	129.8	-45.2	49.1	8.7	46.6	19.0	8.8
DT02	7/7	354/68	127.2	21.8	130.8	-29.2	19.8	13.9	40.6	6.0	11.4
DT03	5/6	354/68	130.9	24.4	131.2	-25.0	33.1	13.5	39.2	3.3	10.6
DT04	6/6	354/68	132.6	13.7	141.7	-30.4	107.2	6.5	48.8	356.7	5.4
DT05	6/7	161/81	189	-27.4	134.6	-28.9	389.3	3.4	43.2	2.5	2.8
DT06	7/7	161/81	180.9	-20.3	142.6	-21.7	109.3	5.8	45.6	350.6	4.4
DT07	6/9	161/81	169.4	-7.0	155.3	-9.3	26.3	13.3	47.3	330.1	9.5
DT08	8/9	161/81	180.3	-19.2	143.8	-21.1	139.9	4.7	46.1	349.0	3.6
DT09	7/7	161/81	181.2	-13.7	149.8	-21.7	633.6	2.4	50.0	342.3	1.8
DT10	6/9	32/28	133.5	-14.6	136.7	-42.1	31.6	11.7	50.4	10.7	11.3
DT11	6/8	32/28	110.3	5.2	109.4	-22.2	18.9	15.8	22.1	18.3	12.2
DT12	8/9	32/28	127.1	-13.5	128.6	-41.4	23.8	11.6	44.0	16.4	11.1
DT13	6/10	32/28	110.7	16.6	110.9	-10.9	30.6	12.3	19.3	12.3	8.9
DT14	6/8	32/28	110.7	13.3	110.6	-14.2	58.9	8.8	20.2	13.9	6.4
DT15	6/10	32/28	124.8	0.3	125.1	-27.6	27.1	13.1	35.8	9.7	10.6
DT16	7/10	32/28	119.6	1.8	119.3	-26.2	18.3	14.5	31.0	13.3	11.5
DT17	13/13	32/28	132.4	-5.2	134.3	-32.6	13.3	11.8	44.5	5.2	10.0
DT18	8/9	32/28	111.6	-7.1	109.5	-34.5	29.3	10.4	26.8	24.6	9.0
DT19	10/10	32/28	137.7	-12.4	141.9	-39.2	78.1	5.5	52.8	3.3	5.1
DT20	11/12	32/28	141.9	-2.1	144.7	-28.3	13.1	13.1	49.8	352.1	10.6
DT21	9/10	32/28	133.4	-15.1	137.0	-42.4	24.1	10.7	50.7	10.8	10.3
Mean [a]	155/183	21 sites	137.7	-4.2	131.7	-29.0	26.2	6.3	41.5	5.4	5.8
**Upper member of the Dahongshan Formation** (~281 Ma) volcanic rocks (LT01-03 and 05-10) and sandstones (LT 04 and 11-15)											
GPS: 41.21°N, 112.20°E (LT01-03); GPS: 41.23°N, 112.20°E (LT04-15)											
LT01	10/11	163/31	181.7	-33.1	159.3	-37.3	34.3	8.4	63.2	339.2	7.6
LT02	10/10	163/31	178.1	-31.3	157.7	-34.1	52.1	6.8	60.5	339.1	5.9
LT03	8/9	163/31	186.5	-29.1	166.4	-36.6	211.5	3.8	66.2	325.2	3.4
LT04	9/10	121/35	176.5	-9.2	166.8	-36.7	55.2	7.0	66.4	324.5	6.3
LT05	8/10	121/35	173.5	-15.0	160.2	-40.7	103.4	5.5	65.5	341.0	5.2
LT06	10/11	121/35	171.8	-13.6	159.1	-38.7	198.0	3.4	63.8	340.8	3.1
LT07	12/15	121/35	178.2	-22.3	160.9	-49.2	139.0	3.7	71.0	352.4	4.0
LT08	6/10	140/20	188.6	-35.7	174.8	-49.1	214.2	4.6	78.0	314.4	4.9
LT09	10/10	140/20	172.8	-26.9	161.8	-36.2	102.4	4.8	63.8	333.9	4.3
LT10	7/10	131/58	167.8	-1.0	151.7	-31.1	70.0	7.3	55.5	345.5	6.1
LT11	9/10	131/58	177.5	6.0	165.5	-33.8	174.5	3.9	64.1	325.1	3.4
LT12	11/11	131/58	184.4	0.4	166.9	-42.6	154.6	3.7	70.2	329.6	3.6
LT13	8/9	131/58	181.4	12.9	174.4	-31.2	45.44	8.3	65.1	305.0	6.9
LT14	8/9	121/39	173.9	-7.4	162.7	-36.7	50.3	7.9	64.6	332.6	7.1
LT15	7/10	121/39	174.9	-7.6	163.8	-37.3	234.9	4.0	65.4	331.0	3.6
Mean [b]	133/155	15 sites	177.6	-14.3	163.3	-38.2	126.0	3.4	66.0	332.8	3.3
**Member 1 of the Qingfengshan Formation** (~281 Ma) red siltstones *(GPS: 42.47°N, 119.30°E)*											
AQ01	7/8	170/26	1.9	23.8	349.5	26.4	121.4	5.5	60.1	320.0	4.4
AQ01*					349.5	35.2	89.7	6.4	65.3	323.5	5.6
AQ02	7/8	170/26	358.5	19.7	348.6	21.3	109.3	5.8	57.1	320.2	4.4
AQ02*					348.6	28.9	85.9	6.5	61.2	322.6	5.3
AQ03	10/10	170/26	10.6	26.2	356.1	32.4	181.0	3.6	64.9	308.0	3.1
AQ03*					356.1	42.0	172.7	3.7	71.5	310.5	3.6
AQ04	10/10	170/26	4.3	25.5	350.8	29.0	90.7	5.1	61.9	318.5	4.2
AQ04*					350.8	38.2	104.2	4.8	67.6	322.4	4.4
AQ05	7/10	170/26	5.2	25.4	351.6	29.3	89.9	6.4	62.3	316.8	5.2
AQ05*					351.6	38.7	98.5	6.1	68.2	320.6	5.6
AQ06	5/9	170/26	10.5	23.9	357.3	30.3	572.7	3.2	63.7	305.1	2.7
AQ06*					357.3	39.8	533.4	3.3	70.0	306.5	3.1
AQ07	9/9	83/13	168.3	-9.8	168.0	-22.8	121.0	4.7	-57.6	141.7	3.6
AQ07*					168.0	-30.7	91.5	5.4	-62.1	144.6	4.5
AQ08	8/10	83/13	165.4	-20.6	164.5	-33.5	25.4	11.2	-62.5	152.7	9.6
AQ08*					164.5	-42.3	20.6	12.5	-67.9	159.7	12.1
AQ09	5/8	83/13	165.1	-25.3	163.9	-38.2	71.7	9.1	-65.0	157.0	8.3
AQ09*					163.9	-48.0	82.7	8.5	-71.3	168.5	9.0
AQ10	6/8	83/13	166.6	-21.9	165.8	-34.8	139.1	5.7	-63.8	151.0	5.0
AQ10*					165.8	-44.8	164.0	5.2	-70.2	159.8	5.2

AQ11	6/10	83/13	166.1	-18.3	165.3	-31.2	98.0	6.8	-61.5	149.8	5.7
AQ11*					165.3	-40.7	98.3	6.8	-67.3	156.3	6.4
AQ12	10/11	83/13	174.4	-14.7	174.5	-27.7	63.7	6.5	-61.8	130.6	5.2
AQ12*					174.5	-36.3	51.8	7.2	-67.2	132.7	6.4
AQ13	9/13	83/13	171.6	-18.4	171.4	-31.4	175.2	3.9	-63.5	137.9	3.3
AQ13*					171.4	-40.9	160.4	4.1	-69.7	142.4	3.9
AQ14	6/7	83/13	172.5	-16.5	172.4	-29.5	154.9	5.4	-62.6	135.2	4.4
AQ14*					172.4	-38.8	144.2	5.6	-68.5	138.7	5.1
AQ15	8/8	83/13	176.5	-14.9	176.8	-27.9	73.6	6.5	-62.2	125.9	5.3
AQ15*					176.8	-37.0	81.5	6.2	-68.0	127.3	5.6
Mean [c]	112/135	15 sites	175.7	-20.5	170.5	-29.8	196.8	2.7	62.3	319.4	2.4
Mean*[d]					170.5	-38.9	183.6	2.8	68.1	323.6	2.5
Mean[b+d]	245/290	30 sites	176.7	-17.5	166.9	-38.6	129.7	2.3	67.1	328.4	2.1

Note: n/N, number of samples used to calculate mean/total samples demagnetized; Strike/Dip, right hand strike/dip of the strata; D/I, declination/inclination; k, the precision parameter; α_{95}, the radius that the mean direction lies within 95% confidence; Plat/Plon, latitude/longitude of paleopoles; dp/dm, semi-axes of elliptical error of the pole at a probability of 95%; *, the results after E/I correction.

[a] (1) fold test is positive at the 95% confidence[15]: Ks/Kg = 3.24 > F(2*(n2−1),(n1−1)) at 5% and 1% point = 1.69 and 2.11; (2) fold test is also positive at the 95% and 99% confidence[16], critical Xi at 95% =5.34, at 99%=7.48; Xi2 IS=14.05, Xi2 TC=1.64; (3) a random distribution test yielded R = 3.39 (n = 9), which is significantly less than the critical R-values of 4.76 and 5.61 at the 95% and 99% confidence levels[17] that are indicative of a positive conglomerate test.

[b] (1) fold test is positive at the 95% and 99% confidence[15]: Ks/Kg = 4.92 > F(2*(n2−1),(n1−1)) at 5% and 1% point = 1.88 and 2.47, respectively; (2) fold test is also positive at the 95% and 99% confidence[16], critical Xi at 95% =4.51, at 99%=6.31; Xi1 IS=1.07, Xi2 TC=1.00; Xi2 IS=11.96, Xi2 TC=1.13; (3) a random distribution test yielded R = 2.21 (n = 11), which is significantly less than the critical R-values of 5.29 and 6.25 at the 95% and 99% confidence levels[17] that are indicative of a positive conglomerate test.

[c] (1) fold test is positive at the 95% and 99% confidence[15]: Ks/Kg = 2.79 > F(2*(n2−1),(n1−1)) at 5% and 1% point = 1.88 and 2.47, respectively; (2) fold test is also positive at the 95% and 99% confidence[16], critical Xi at 95% =4.51, at 99%=6.31; Xi1 IS=8.94, Xi2 TC=1.56; Xi2 IS=12.39, Xi2 TC=2.30; (3) reversal test[18], angle between the two averages γ=3.7°< $\gamma_{critical}$=5.5° indicates a B class result.

[b+d] (1) fold test is positive at the 95% and 99% confidence[15]: Ks/Kg = 3.55 > F(2*(n2−1),(n1−1)) at 5% and 1% point = 1.55 and 1.86, respectively; (2) fold test is also positive at the 95% and 99% confidence[16], critical Xi at 95% =6.37, at 99%=9.01; Xi1 IS=8.32, Xi2 TC=0.22; Xi2 IS=21.01, Xi2 TC=4.19; (3) reversal test[18], angle between the two averages γ=5.5°< $\gamma_{critical}$=5.6° indicates a B class result. (4) In tilt-corrected, the VGPs between the two strata pass a significance test[19]: LT01-15: xMin/xMax=-0.41/-0.32, yMin/yMax=0.15/0.22, zMin/zMax=-0.93/-0.89; AQ01-15: xMin/xMax=-0.33/-0.27, yMin/yMax=0.18/0.26, zMin/zMax=-0.94/-0.92.

Supplementary Table 3.

The Late Carboniferous-Early Triassic paleopoles from the North China Block, South China Block and North Qiangtang Block.

Age (Ma)	Lithology	N [n]	Plat (°N)	Plong (°E)	A_{95} (°)	PaLat (°N)	Criterion (R)	References
				**North China Block**				
*250	C	7 studies	60.8	13.4	5.8		12345R7(7)	Zhou et al.[6]
255	V	14[109]	54.2	20.2	3.3	28.6	123F5R7(7)	Ren et al.[2]
260	C	4[18]	47.0	359.2	4.2	13.9	1-3-5R7(6)	Ma et al.[5]
*260		4[18]	51.7	10.1	4.2	22.2		Ma et al.[5]
265	V+C	11	53.1	336.3	8.8	10.7	123-5R7(6)	Zhao et al.[4]
275	V	24	66.2	328.1	4.1	20.9	123F5-7(6)	Zhang et al.[3]
**281**	**V+C**	**30[245]**	**67.1**	**328.4**	**2.1**	**21.8**	**123FC5R7(7)**	**This study**
**290**	**V**	**21[155]**	**41.5**	**5.4**	**5.8**	**12.8**	**123FC5-7(6)**	**This study**
305	L	4	33.3	10.2	16.7	9.6	1-3-5-7(4)	Huang et al.[20]
				**South China Block**				
260	V+C+L	22 studies	48.6	226.4	4.6	6.9		Huang et al.[21]
270	V	6 studies	53.8	248.1	4.3	0.5		Huang et al.[21]
278	L	7[76]	65.3	265.2	5.8	5.7	123-5-7(5)	Wu et al.[22]
298	L	4	21.5	224.6	12.3	-7.5	1-3-5R7(5)	Lin[23]
				**North Qiangtang Block**				
256	V+C	9	11.9	199.7	9.3	-7.5	123F5-7(6)	Cheng et al.[24]
259	V	28	13.6	2.4	5.6	-7.9	123F5-7(6)	Ma et al.[25]
265	L	5	-1.0	204.2	14.6	-18.8	123F5-7(6)	Cheng et al.[24]
280	V+L	5	31.7	226.8	12.5	-11.7	123F5-7(6)	Cheng et al.[26]
297	V	14	21.7	232.9	8.9	-23.0	123F5-7(6)	Song et al.[27]

Note: N[n] = number of sites [samples] used for pole determination; Plat and Plon = latitude and longitude of the pole; A_{95} = the radius that the mean pole lies within 95% confidence; PaLat = paleolatitude (reference point: 42.5°N, 119.5°E for North China, 28.0°N, 113.0°E for South China, and 34.0°N, 92.0°E for North Qiangtang); Criterion (R, numbers of the criteria met), reliability criteria 1–7 from Meert et al.[28] [1 = Well-determined rock age and a presumption that magnetization is the same age; 2 = Techniques and Statistical analysis; 3 = Evaluation of remanence carriers; 4 = Field tests that constrain age of magnetization (F, the positive fold test; C, the positive conglomerate test); 5 = Structural control, and tectonic coherence with craton or block involved; 6 = the presence of magnetic reversals; 7 = no resemblance to younger poles by more than a period based on overlapping A_{95}]; "-" = failed to meet this criterion. C, clastic rocks; L, limestones; V, volcanic rocks. Asterisks represent results corrected for inclination flattening with a flattening factor f = 0.6.

Supplementary Table 4.

The error of paleolatitude and age.

Reference	Age_mean (Ma)	Age_error (Ma)	Mean_Lat (°N)	Low_Lat (°N)	High_Lat (°N)	Estimate error
North China Block						
Ren et al.[2]	255.95	1.15	28.54	25.93	31.33	0.9174
Ma et al.[5]	259.0	1.0	22.26	19.16	25.62	1.0802
Zhao et al.[4]	265.0	5.0	10.70	4.58	17.54	2.1978
Zhang et al.[3]	274.5	8.5	20.92	17.93	24.16	1.0416
This study*	280.6	0.7	21.48	19.03	24.09	0.8450
This study	289.95	1.75	12.84	8.75	17.29	1.4653
Huang et al.[20]	302.95	4.05	9.61	-1.75	23.42	4.2311
South China Block						
Huang et al.[21]	260.0	10.0	6.89	3.67	10.26	1.1200
Huang et al.[21]	270.0	10.0	0.55	-2.51	3.61	1.0216
Wu et al.[22]	278.9	4.4	5.76	1.70	9.99	1.3857
Lin[23]	298.8	5.3	-7.47	-17.04	1.05	3.0578
North Qiangtang Block						
Cheng et al.[24]	256.0	4.0	-7.53	-14.59	-1.05	2.2639
Ma et al.[25]	259.1	1.4	-7.89	-12.04	-3.97	1.3501
Cheng et al.[24]	265.5	5.5	-18.83	-31.79	-8.96	3.9262
Cheng et al.[26]	279.5	19.5	-11.7	-21.83	-2.86	3.2084
Song et al.[27]	296.9	1.9	-23.05	-30.70	-16.66	2.4065

Note: Lat, Latitude; Estimate error, the distribution error of latitudes calculated by mean, low and high latitudes. *, data from upper member of Dahongshan Formation.

Supplementary Table 5.

The estimated latitudinal rate of plate motion.

Stage	age interval (Ma)	Latitudinal motion rate							
		raw rate (°/My)	mean (°/My)	mode (°/My)	**median (°/My)**	2.5 percentile	97.5 percentile	**Median (cm/yr)**	95% confidence interval
					North China Block				
Stage 1	259.0-255.95	-2.05902	-2.58521	-1.9988	**-2.036**	-4.3783	-1.0008	**-22.80**	(-48.60, -11.11)
Stage 2	265.0-259.0	-1.92667	-0.64863	-1.0605	**-1.514**	-8.7646	0.808	**-21.14**	(-97.29, 8.97)
Stage 3	274.5-265.0	1.075789	1.221367	0.396	**0.73865**	0.0449	6.7424	**11.48**	(0.50, 74.84)
Stage 4	280.6-274.5	0.091803	0.123343	-0.054	**0.0735**	-1.53065	1.77734	**0.82**	(-17.00, 19.73)
Stage 5	289.95-280.6	-0.92406	-0.96496	-0.9404	**-0.9219**	-1.3789	-0.551	**-10.23**	(-15.31, -6.12)
Stage 6	302.95-289.95	-0.24846	-0.27072	-0.4556	**-0.25025**	0.9973	0.44	**-2.75**	(11.07, 4.88)
					South China Block				
Stage 1	270.0-260.0	-0.634	-0.24928	-0.2434	**-0.556**	-5.8594	4.3685	**-6.17**	(-65.04, 48.49)
Stage 2	278.9-270.0	0.585393	0.35519	0.4195	**0.5411**	-2.8982	4.7017	**6.01**	(-32.17, 52.19)
Stage 3	298.8-278.9	-0.66482	-0.6674	-0.5875	**-0.665**	-1.1692	0.3295	**-7.38**	(-12.98, 3.66)
					North Qiangtang Block				
Stage 1	259.1-256.0	-0.11613	-0.07844	-0.3449	**-0.0758**	-5.3698	4.7054	**-0.84**	(-59.61, 52.23)
Stage 2	265.5-259.1	-1.70938	-0.13206	-1.5582	**-1.6763**	-8.3868	-0.2317	**-18.61**	(-93.09, -2.57)
Stage 3	279.5-265.5	0.509286	1.063796	0.2125	**0.4186**	-3.912	5.229	**4.65**	(-43.42, 58.04)
Stage 4	296.9-279.5	-0.6523	-0.13277	-0.2963	**-0.6063**	-5.0448	2.4909	**-6.73**	(-56.00, 27.65)

Note: raw rate was directly calculated using the mean latitude and mean age. The rest rates are values obtained from the 10000 times Monte Carlo simulation. Mean, arithmetic mean value; mode, the most frequently occurring data in a set of data; median, the data in the middle of a sequence of data. The data are calculated by the raw data in Supplementary Table 4. Negative values represent northward movements; positive values represent southward movements.

SUPPLEMENTARY REFERENCES

1 Wu, H., Zhu, R., Liu, C. & Chang, C. Paleomagnetic observations in North China Block: From Late Paleozoic to Triassic. *Acta Geophysica Sinica* **33**, 694-701 (1990).

2 Ren, Q. *et al.* New middle–late Permian paleomagnetic and geochronological results from Inner Mongolia and their paleogeographic implications. *Journal of Geophysical Research: Solid Earth* **125**, e2019JB019114 (2020).

3 Zhang, D. *et al.* Permian paleogeography of the eastern CAOB: Paleomagnetic constraints from volcanic rocks in central eastern Inner Mongolia, NE China. *Journal of Geophysical Research: Solid Earth* **123**, 2559-2582 (2018).

4 Zhao, X., Robert S, C., Zhou, Y., Wu, H. & Wang, J. New paleomagnetic results from northern China: collision and suturing with Siberia and Kazakhstan. *Tectonophysics* **181**, 43-81 (1990).

5 Ma, X., Xing, L., Yang, Z., Xu, S. & Zhang, J. Paleomagnetic study since late Paleozoic in the Ordos basin. *Acta Geophysica Sinica* **36**, 68-79 (1993).

6 Zhou, T. *et al.* Paleomagnetic inclination shallowing in lower Triassic Liujiagou Formation from Qinshui basin, north China block. *Acta Scientiarum Naturalium Universitatis Pekinensis* **54**, 521 (2018).

7 Embleton, B. J. J., Mcelhinny, M. W., Ma, X., Zhang, Z. & Zheng, X. L. Permo-Triassic magnetostratigraphy in China: the type section near Taiyuan, Shanxi Province, North China. *Geophysical Journal of the Royal Astronomical Society* **126**, 382-388 (2018).

8 Tan, X., Fang, D., Yuan, Y., Fan, S. & Bao, J. Paleomagnetic study on red beds, Liujiaogou Formation (TRL) from Taoyuan, Yaoqu, Jixian, Shanxi Province. *Acta Geophysics Sinica* **34**, 736-743 (1991).

9 Fang, D. *et al.* Tectonic implications of Triassic and Jurassic paleomagnetic results from Ningwu basin, Shanxi Province. *Kexue Tongbao* **2**, 133-135 (1988).

10 Yang, Z. *et al.* Paleomagnetic results from Triassic sections in the Ordos basin, north China. *Earth and Planetary Science Letters* **104**, 258-277 (1991).

11 Huang, B., Shi, R., Wang, Y. & Zhu, R. Palaeomagnetic investigation on Early—Middle Triassic sediments of the North China block: a new Early Triassic palaeopole and its tectonic implications. *Geophysical Journal International* **160**, 101-113 (2005).

12 Tauxe, L. & Kent, D. V. A simplified statistical model for the geomagnetic field and the detection of shallow bias in paleomagnetic inclinations: was the ancient magnetic field dipolar? (2004).

13 Wu, Q. *et al.* High-precision U-Pb age constraints on the Permian floral turnovers, paleoclimate change, and tectonics of the North China block. *Geology* **49**, 677-681 (2021).

14 Shen, B. *et al.* Carboniferous and Permian integrative stratigraphy and timescale of North China Block. *Science China Earth Sciences* **65**, 983-1011 (2022).

15 McElhinny, M. Statistical significance of the fold test in palaeomagnetism. *Geophysical Journal International* **8**, 338-340 (1964).

16 McFadden, P. A new fold test for palaeomagnetic studies. *Geophysical Journal International* **103**, 163-169 (1990).

17 Watson, G. A test for randomness of directions. *Geophysical Supplements to the Monthly Notices of the Royal Astronomical Society* **7**, 160-161 (1956).

18 McFadden, P. & McElhinny, M. Classification of the reversal test in palaeomagnetism. *Geophysical Journal International* **103**, 725-729 (1990).

19 Tauxe, L. *Essentials of paleomagnetism*. (Univ of California Press, 2010).

20 Huang, B., Otofuji, Y.-i., Zhu, R., Shi, R. & Wang, Y. Paleomagnetism of Carboniferous sediments in the Hexi corridor: its origin and tectonic implications. *Earth and Planetary Science Letters* **194**, 135-149 (2001).
21 Huang, B. *et al.* Paleomagnetic constraints on the paleogeography of the East Asian blocks during Late Paleozoic and Early Mesozoic times. *Earth-science reviews* **186**, 8-36 (2018).
22 Wu, H. *et al.* Paleomagnetic study of Paleozoic to Mesozoic rocks from the Xingshan-Zigui section of the Yangtze Block, Hubei, China. *Sci. China Ser. D Earth Sci* **29**, 144-154 (1999).
23 Lin, J. *The apparent polar wander paths for the north and south China blocks, 248 pp*, Ph. D. thesis, Univ. of Calif., Santa Barbara, (1984).
24 Cheng, X. *et al.* Paleomagnetic data from the Late Carboniferous-Late Permian rocks in eastern Tibet and their implications for tectonic evolution of the northern Qiangtang-Qamdo block. *Science China Earth Sciences* **56**, 1209-1220 (2013).
25 Ma, Y. *et al.* Paleomagnetic constraints on the origin and drift history of the North Qiangtang terrane in the Late Paleozoic. *Geophysical Research Letters* **46**, 689-697 (2019).
26 Cheng, X. *et al.* Paleomagnetic results of late Paleozoic rocks from northern Qiangtang block in Qinghai-Tibet plateau, China. *Science China Earth Sciences* **55**, 67-75 (2012).
27 Song, P., Ding, L., Li, Z., Lippert, P. C. & Yue, Y. An early bird from Gondwana: Paleomagnetism of Lower Permian lavas from northern Qiangtang (Tibet) and the geography of the Paleo-Tethys. *Earth and Planetary Science Letters* **475**, 119-133 (2017).
28 Meert, J. G. *et al.* The magnificent seven: a proposal for modest revision of the quality index. *Tectonophysics* **790**, 228549 (2020).
